# Stress during the COVID-19 pandemic - impact on neuroplasticity

**DOI:** 10.1192/j.eurpsy.2022.2090

**Published:** 2022-09-01

**Authors:** A.C. Bondar, A.-G. Buciuc

**Affiliations:** 1 Titu Maiorescu University, Psychiatry, Bucharest, Romania; 2 “Prof. Dr. Al. Obregia” Clinical Hospital of Psychiatry, General Psychiatry, Bucharest, Romania

**Keywords:** Depression, Stress, Neuroplasticity, Anxiety

## Abstract

**Introduction:**

The world’s population has been exposed to traumatic events and high levels of stress due to the ongoing COVID-19 outbreak. Stress is known currently as a universal experience, but the concept was first defined in 1936 by Hans Selye. It has been shown that stress is associated with impairments in neuroplasticity (e.g. neuronal atrophy and synaptic loss in the hippocampus, prefrontal cortex) and has a crucial role in almost all mental disorders.

**Objectives:**

In this paper we aim to highlight the recent theoretical and experimental advances in neuroscience regarding stress induced neuroplasticity.

**Methods:**

We analyzed scientific literature written in English and published between 2019-2021. We used the electronic portal PubMed-NCBI.

**Results:**

In the last few years, molecular and cellular studies on animal models of stress related and stress-induced psychopathologies revealed alterations in gene expression, micro ARNs expression, as well as in intracellular signaling pathways that mediate the stress induced adaptations. These findings have led to new theories regarding depression and anxiety in the molecular neurobiology field. It has been shown that stress reduces BDNF expression inducing neuronal atrophy in various brain areas. Contrastingly, other studies have demonstrated that chronic antidepressant treatment increases BDNF expression. Furthermore, a crucial role has been assigned to miRNAs in the development of chronic stress-induced depression-like behavior and neuroplasticity.

**Conclusions:**

We hope that this paper will increase interest in the field of stress induced cellular and molecular changes. More research needs to be pursued in order to achieve a deeper understanding of the pathophysiology of stress-induced mental disorders.

**Disclosure:**

No significant relationships.

